# Burning Mouth Syndrome due to Television Moans, an Enigma for Oral Physician: Treatment with Counseling

**DOI:** 10.5681/joddd.2014.022

**Published:** 2014-06-11

**Authors:** Deepak Gupta, Soheyl Sheikh, Shambulingappa Pallagatti, Kartikaya Kasariya, Amit Buttan, Maqul Gupta

**Affiliations:** ^1^Senior Lecturer, Department of Oral Medicine and Radiology, M.M. College of Dental Sciences and Research, Mullana, Ambala, Haryana, India; ^2^Professor and Head of the Department of Oral Medicine and Radiology, M.M. College of Dental Sciences and Research, Mullana, Ambala, Haryana, India; ^3^Senior Lecturer, Department of Conservative Dentistry and Endodontics, M.M. College of Dental Sciences and Research, Mullana, Ambala, Haryana, India; ^4^Senior Lecturer, Department of Periodontics, Sardar Patel Post Graduate Institute of Dental and Medical Sciences and Research, Lucknow, Uttar Pra-desh, India; ^5^Physician, DNB Anesthesia, Kasturba Hospital, Daryaganj, New Delhi, India; ^6^Family Physician, Appolo Hospital, Ludhiana, Punjab, India

**Keywords:** Burning mouth syndrome, counseling, orofacial pain, psychological disorders

## Abstract

Burning mouth syndrome (BMS) is a relatively common disease that can severely affect the quality of life of the patient. It causes chronic orofacial pain or oral burning sensation even in the absence of any detectable organic cause. The etiology of BMS is complex and multifactorial. It has been associated with menopause, trigger events and even genetic polymorphisms. Although its etiology remains unclear, there is still much evidence that psychological elements like stress, anxiety or depression do play a significant role. There are several studies in the literature which only report the association of BMS with psychological factors. But to the best of our knowledge, there is no such case reported in the literature which has actually highlighted the management of such a case with psychogenic elements involved. In this case report, apart from discussing the role of psychological factors, the treatment of BMS with emphasis on counseling is also emphasized. Further, it is of interest to know that such patients with psychologically induced burning mouth syndrome have to be evaluated to their deepest details. Even their commonly overlooked gestures and habits like watching a particular television soap opera may be involved in their disease process. It can be concluded that psychological counseling in general dental practice can provide an effective cure for chronic oral burning sensation with psychological factors involved.

## Introduction

Burning mouth syndrome (BMS) is a chronic oral dysesthesia characterized by a burning sensation of the oral cavity with clinically normal mucosa and absence of any detectable organic cause.^1-6^ There are no abnormal laboratory findings.^4^ The burning sensation is of sufficient severity to attract clinical attention. It may be felt as a continuous or intermittent discomfort, which may affect almost all parts of the oral cavity.^4^ This condition often has a negative ef-fect on the health-related quality of life in patients.^7-9^

Epidemiological studies on BMS have estimated a prevalence rate of 2.6 5.1%.^5^ It predominantly affects post-menopausal women in their fourth and fifth decades of life, with an overall 7:1 female-to-male ratio.^4^ The burning has been reported to be of moderate or severe intensity and may vary throughout the day.^7^ It may occur due to local, systemic and psychogenic factors.^5,6^ Although the literature is replete with debates on BMS, the etiology still remains unclear. Henceforth it is difficult to establish any particular treatment protocol. Several authors have suggested the role of stressful life events and long-term social problems in the etiology of BMS.^5^

Although the literature is being flooded with statements of association of BMS with psychological elements, the general dental practitioners still are not competent enough to recognize such cases. If psychological factors are involved in such patients, it is usually beneficial for the patient to receive antidepressant drugs. Hence the dental and medical literature fairly falls short of management of such cases with psychological counseling.

Although stress has a profound impact on daily life, it remains undiagnosed and undertreated. This proves that there is a significant gap in the application of the current knowledge.

Further, clinical psychology, as well as medical psychiatry, is often added as a part or separate subject in the undergraduate dental curriculum in various dental schools all around the world. This proves that dental graduates must possess the knowledge to recognize, as well as treat, such patients. But no such case treated with psychological counseling has been reported in the dental literature to date to the best of our knowledge. Therefore, the approach towards such patients and their management with counseling is discussed.

## Case Description

A 55-year-old female patient presented with an 8-month old history of burning sensation in the oral cavity. It was more pronounced at the corners of the mouth, lower labial mucosa, vestibule and at the tip of the tongue. Burning sensation was continuous and severe, with disturbed sleep and difficulty in eating food. The patient’s past medical history was non-contributory except for the consumption of anti-arthritic drugs for the past three years. She had consulted a variety of medical practitioners. Prescriptions involved medications including antibiotics, analgesics and antacids. However, only a short-term relief was reported with topical and systemic steroids.

**Figure 1.**
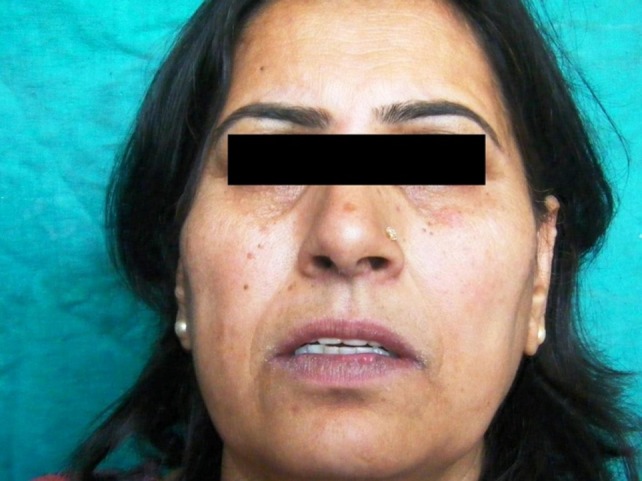
Dryness and cracking of lips with discoloration present at the lower lip.

**Figure 2.**
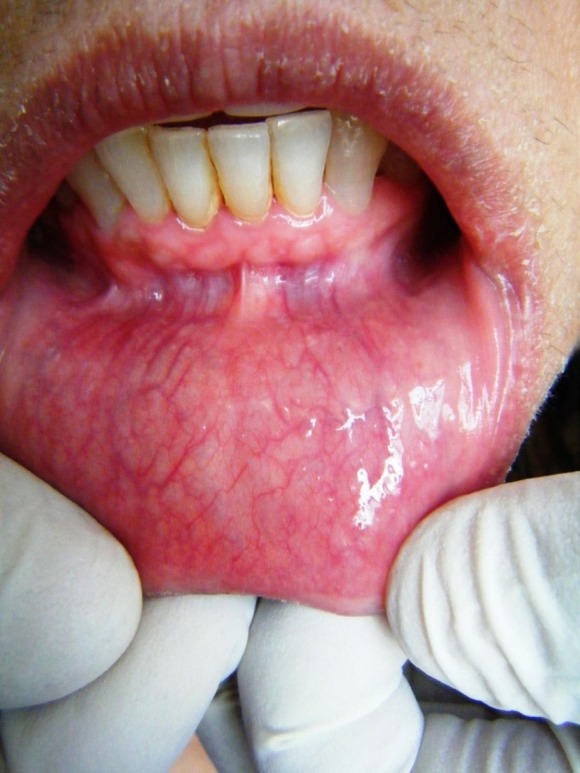
Mild erythema of the lower labial mucosa with no other clinically significant sign.

**Figure 3.**
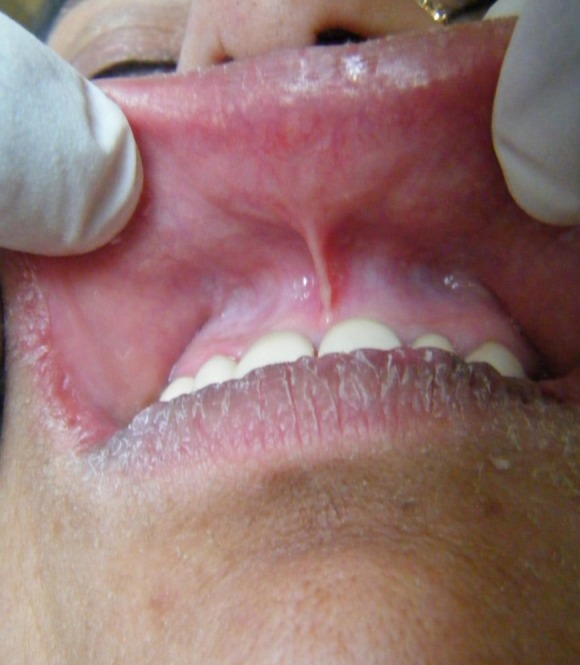
Upper labial mucosa.

There was dryness and cracking of both lips with discoloration present (Figure 1) at the lower lip. Intraorally, there was burning sensation with no significant clinically detectable finding. However, detailed exploration revealed the slight reddening of the lower labial mucosa (Figure 2) as compared to the upper one (Figure 3) with no other clinically significant sign. Redness was interspersed with normal mucosa. Presence of calculus in the interproximal areas of mandibular anterior teeth was noticed (Figure 2). In addition, the marginal and the attached gingiva were also erythmatous with no significant bleeding on probing. There was no discontinuity of the mandibular labial mucosa. Intraoral palpation exacerbated the burning sensation at the mandibular labial mucosa as well as the gums in the anterior region. Burning sensation was bilaterally symmetrical. All the rest of oral findings were non-contributory. Suspecting lichenoid reaction, the patient was advised to stop anti-arthritic medication for one week. No relief was reported. Considering nutritional deficiency as a causative factor, the patient was subjected to various laboratory investigations. Meanwhile the patient was also advised not to consume anti-arthritic medication for some more time and a follow-up was scheduled after one week. But there was no relief.

Routine blood investigation, blood sugar level, thyroid and lipid profile, serum estrogen level, vitamin B_12_(with B_1_, B_2_ and B_6_), folate, serum iron, TIBC, ferritin levels, and percentage of transferrin saturation were carried out. All the values were within the normal range.

On the basis of insignificant laboratory and clinically findings, a working diagnosis of burning mouth syndrome was achieved. Superinfection with *Candida* was suspected at the vermillion border of the lips. Local application of candid gel at the lips was advised but there was no relief. Further, 4-mg triamcinolone tablets t.i.d. and local application of ointment kenakort (for oral use) were advised. After two weeks the patient presented with 50% relief in the burning sensation. The dryness of the lips disappeared. The discoloration and reddening of lower labial mucosa was also reduced.

**Figure 4.**
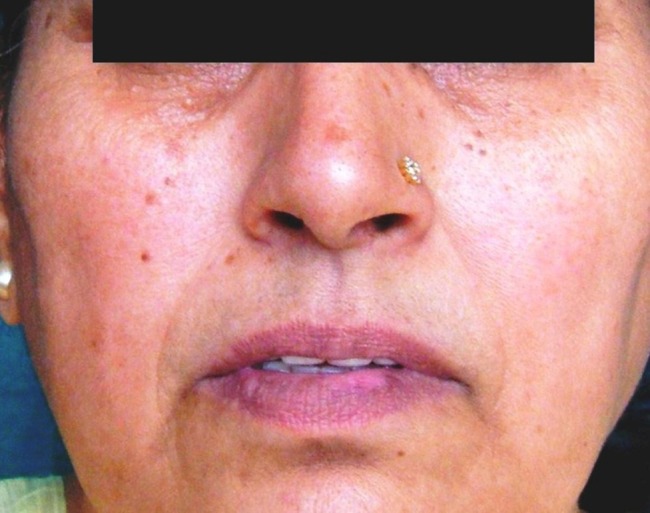
Discoloration of the lower lip started re-appearing.

The medication was continued and the patient’s condition further improved. Owing to the improvement in the patient’s signs and symptoms, triamcinolone tablet (4 mg) was tapered to twice and then to once a day with a two-week gap. In contrast to the previous findings, burning sensation increased this time. Discoloration of lower lip (Figure 4) and redness on the lower labial mucosa reappeared. Dosage was increased to twice a day but there was no significant improvement. Further the patient complained of disturbed sleep occasionally. Alprazolam tablet (0.25 mg) was added to the previous regime. After 2 weeks the burning sensation was reduced.

Detailed history of burning sensation was considered. Patient’s burning sensation was mild after she woke up in the morning and increased as the day progressed to evening. It decreased during the patient’s evening walk and while doing some works like cooking. Her husband reported that she presents with less discomfort and pain whenever she goes on vacations. Clearly, stress was a causative factor.

She was then subjected to Holmes and Rahe stress rating scale.^10^ Her stress score was 249 which according to the scale showed moderate life crisis with more than 50% chances of illness due to stress. The patient was referred to a psychiatrist for opinion. The psychiatrist reported that the patient was suffering from multiple family stresses with reduced sleep. She had a sluggish daily routine. Counseling and anxiolytics were advised.

The patient was then properly inquired about family stress. She admitted to have regular disputes with her daughter-in-law. In addition, burning sensation increased while watching TV sometimes. On questioning, she admitted that she watched soap serials presenting family disputes. A final diagnosis of burning mouth syndrome was arrived at, after which all the medications were tapered off and discontinued.

**Figure 5.**
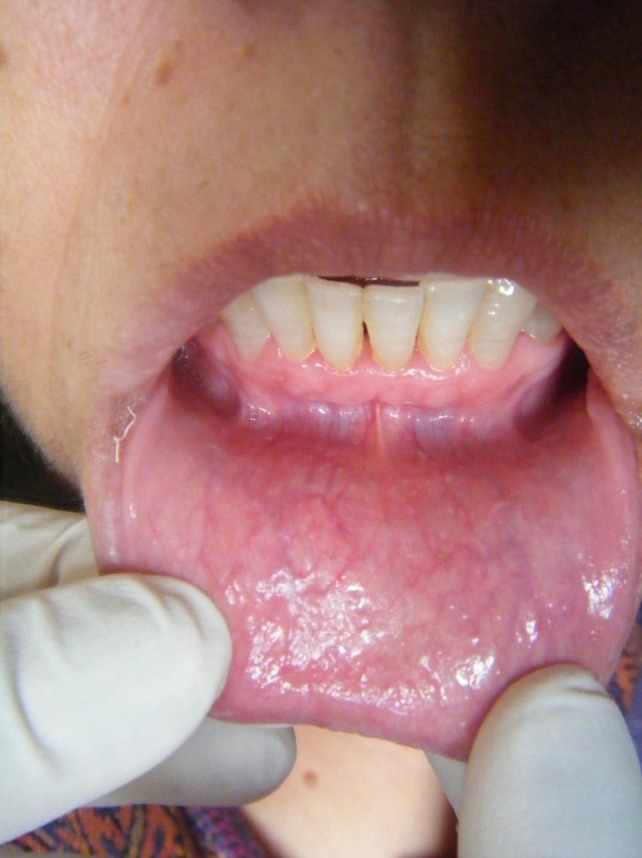
Resolution of redness of the lower labial mucosa.

**Figure 6.**
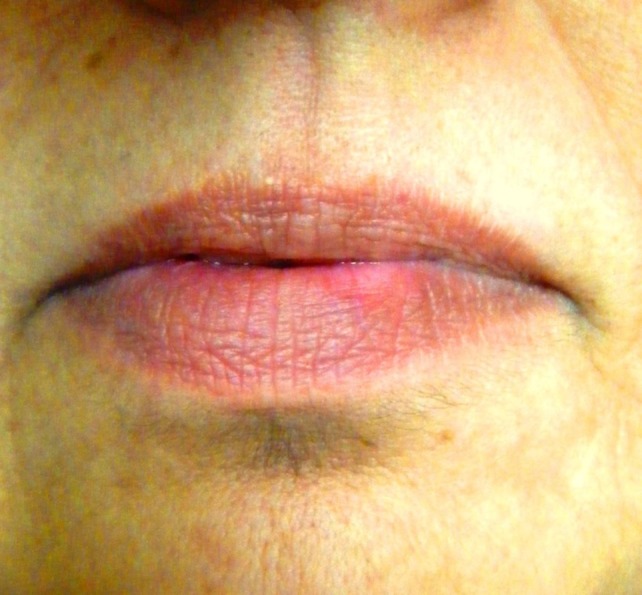
Post-treatment photograph.

The patient and her husband were made aware of the condition and good diet with increased water intake was advised. She was also advised to keep a check on stressful television soap operas, a walk two to three times a day and to keep her busy with some kind of work. After two weeks, the burning sensation had completely disappeared. The redness on lower labial mucosa (Figure 5) and the discoloration on the lower lip were resolved completely (Figure 6). There was no associated difficulty while eating food. The patient was further counseled regarding the situation and was kept on follow-ups.

## Discussion

Although burning mouth syndrome has been described for many years, it still poses problems during diagnosis and treatment.^9,11^ Further, the burning sensation cannot justify the type of lesions if present. Nowadays debate is going on whether psychological alterations in BMS patients are the cause or consequence of this chronic oral pain.^11^

Although BMS cannot be detected clinically, the importance of clinical examination cannot be overlooked. BMS burning is almost always bilateral and symmetrical and does not follow the anatomical distribution of a peripheral sensory nerve.^1,8^ This is an important part of the clinical examination in order to exclude any anatomical distribution to the dysesthesia, which may indicate a neurogenic pain.^8^

Local, systemic and environmental or psychogenic causes must be assessed to elicit the predisposing factors.^1-5^ Assessments of local factors include ill-fitting dentures and allergic response to a variety of restorative materials. Various systemic causes associated with BMS include menopause, deficiency of vitamin B-complex, iron deficiency anemia and prolonged diabetes mellitus.^12,13^ All these systemic diseases need to be ruled out with appropriate blood tests as in this reported case. Somatoform pain disorder has been suggested as a mechanism and psychological, psychosocial and even psychiatric disorders play a demonstrable role in BMS etiology and symptomatology.^1^

Psychological factors could help explain BMS in at least 50% of patients.^9,11^ This can be supported by the study carried out by Komiyama et al, who reported that somatization score was more in BMS patients as compared to trigeminal neuralgia patients.^12^It was also debated that pain intensity and psychosocial scores increased in BMS patients when illness duration was prolonged.^12^ More recently, an enticing hypothesis has been proposed that burning mouth syndrome is associated with an alteration of gonadal, adrenal and neuroactive steroid levels.^14^ A low dose of anxiolytics will usually provide some level of dampening of symptom intensity in such cases. This will aid in confirming the diagnosis and to strengthen the basis of clinical discussion and decisions regarding the ongoing management.

As discussed above, the etiology and diagnosis of BMS is not clear.^15,16^ Therefore, the management approach for such patients is still a matter of controversy. Several authors have reported that BMS patients may require pain control that targets the central nervous system or psychosocial characteristics.^12^ As a result, the treatment of BMS patients involve the management of both physiological and psychological factors.^16^ But it is very hard for the patient to accept psychological disease as a major component of the disease. This major hurdle can be overcome with reinforcement by clinical signs of patient’s stress, anxiety or depression which may include: (a) xerostomia, which can be caused by anxiety; (b) dryness of the inner aspect of the lower lip from minor labial gland hypofunction; (c) scalloping of the lateral lingual margins or roughness of buccal mucosa secondary to habitual pressure against the adjacent teeth; (d) low-grade erythema of the anterior dorsum of the tongue and anterior hard palate as a result of traumatic abrasion against each other; and (f) low-grade linear erythema of the inner aspect of the lip coincident with the edges of the incisor teeth, mainly on the lower lip as in this reported case.^1^

Masato et al in 2006 concluded that BMS was the most frequent diagnosis in the oral medicine consultation clinic for atypical orofacial pain. Somatoform disorder, including pain disorder, was the major diagnosis for the BMS by doctors of psychosomatic medicine. Hence, dentists should have knowledge about somatoform disorders in order to cooperate with doctors of psychosomatic medicine.^17^ It can be concluded that the identification of symptoms, rather than objective clinical or laboratory findings, is often used to assess BMS. Further, the patients must be counseled to accept that the psychogenic elements are related to their symptoms.

## Conclusion

It is of interest that patients with psychologically induced burning have to be evaluated to their deepest details. Even their commonly overlooked gestures and habits like watching a particular television program may be involved in their disease process. It can be concluded that psychological counseling in general dental practice can provide an effective cure for chronic oral burning with psychological factors involved.
